# Large pilot scale cultivation process study of adherent MDBK cells for porcine Influenza A virus propagation using a novel disposable stirred-tank bioreactor

**DOI:** 10.1186/1753-6561-5-S8-P128

**Published:** 2011-11-22

**Authors:** Boris Hundt, Noreen Mölle, Sabine Stefaniak, Ralf Dürrwald, Joerg Weyand

**Affiliations:** 1 IDT Biologika GmbH, Am Pharmapark, D-06861 Dessau-Roßlau, Germany; 2 Sartorius Stedim Biotech GmbH, August-Spindler-Strasse 11, D-37079 Göttingen, Germany

## Introduction

Influenza is one of the major viral diseases and has tremendous importance for human and animal health worldwide. The etiologic agent, Influenza A virus belongs to the family *Orthomyxoviridae* and is a single stranded (-) enveloped RNA virus with round shape and a diameter of 90-130 nm. Its genome consists of 8 segments which code for up to 11 proteins. The major surface proteins and antigenic determinants are hemagglutinin (HA) and neuraminidase (NA). Pigs are of special importance for Influenza. They are susceptible for a number of Influenza A viruses from different species (human, avian, porcine); in the case of multiple infections of swine new and dangerous virus strains can be assembled by reassortment of genes (antigenic shift). The strain which was used in this study is part of a trivalent inactivated vaccine against porcine Influenza (IDT Biologika GmbH).

The cultivation of Influenza virus for the production of vaccines is often still done in embryonated hens eggs, although there are a number of cell lines available which propagate the virus, e. g. VERO or MDCK cells. Egg production processes are limited in terms of capacity whereas cell culture based processes are more flexible and easy to scale up when facing pandemic situations (H5N1 in 2005 and H1N1pan in 2009/10). Currently, adherent MDBK cells are used for the production of porcine Influenza A viruses at commercial scale (IDT Biologika GmbH). This process is performed in roller bottles so far using a biphasic process scheme (cell growth and virus propagation phase) and is thus limited in capacity, yields and process control. The main goal of this study was to explore options for direct transfer of this process into a novel disposable stirred-tank bioreactor (BIOSTAT CultiBag STR, Sartorius Stedim Biotech GmbH) using microcarriers for the attachment of MDBK cells. The advantage of this system is its scalability up to 1000 l, better process control and presumably higher virus yields. The use of disposables has positive effects on costs and efforts but also for cleaning validation in a multi-purpose facility. Based on the results of this study further optimization steps can be done and the implementation of such systems in GMP routine vaccine production can be evaluated.

## Material and methods

For the seed train MDBK cells were cultivated in roller bottles using a minimal essential medium (MEM) supplemented with 5 % y-irradiated fetal bovine serum (FBS) and 1 % L-glutamine (growth medium). This growth medium was also used later for the cultivation in the BIOSTAT CultiBag STR50 system using a y-irradiated bag with implemented stirrer (two 3-blade impeller). The working volume for this experiment was 35 l (70 % of the maximum reactor capacity). The MDBK cells were seeded with 2.0 x 10^5^ cells/ml; a microcarrier concentration of 2 g/l Cytodex^™^ 1 was chosen. The cells were cultivated at 37 °C with 50 rpm stirrer speed, pH-value was controlled with 1 M NaOH to a level of 7.40, pO_2_ was kept at levels > 40 % saturation by pulsing or constant aeration with sterile air. A biphasic process scheme was applied; cell growth phase (0 – 120 h cultivation time) and virus propagation phase (120 – 192 h cultivation time). For infection a media change was performed: cells attached to the microcarriers were allowed to settle down by stopping agitation for 2 h, after two rinsing steps with PBS the cell culture medium was completely exchanged against the same MEM with 1 % L-Glutamin but without FBS (infection medium). Afterwards, a small amount of infection medium containing porcine Influenza A virus/H1N1/strain 2617 originating from WSV (IDT Biologika GmbH) with a moi of 10^-4^ and a trypsine concentration of 40 units/ml was given into the bioreactor. Samples were taken in distinct intervals and aliquoted for offline analytics including cell count with CASY model TT (Roche Diagnostics), further cell analytics for apoptosis and necrosis with a flow cytometer Guava easycyte and respective commercial assays (Millipore GmbH), automated enzymatic assays for quantification of glucose, lactate, glutamine and ammonia levels (YSI 7100 MBS, Yellow Springs Instruments) and quantification of porcine Influenza virus with hemagglutination (HA) and virus titration assay (TCID_50_) according to IDT internal protocols. Online data, especially for temperature, stirrer speed, pH-value, pO_2_ and gassing rate were recorded by the software MFCS/DA (Sartorius Stedim Biotech GmbH).

## Results and discussion

**Figure 1:**
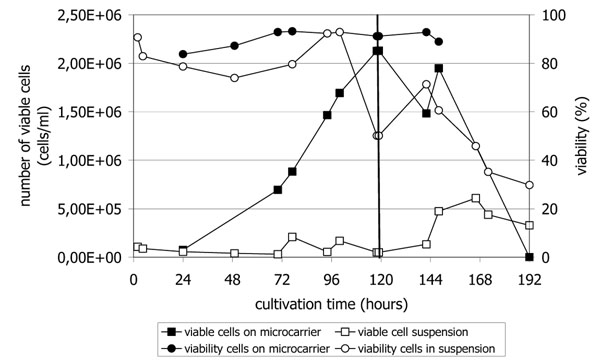
MDBK cell growth and viability for cell populations on microcarriers and in suspension during cultivation in a 50 l scale disposable stirred-tank bioreactor run for propagation of porcine Influenza A/H1N1 (black line indicates virus infection).

After seeding the cells attached rapidly onto the microcarriers; at first analytics after 24 h cells showed a good distribution on the microcarriers with typical morphology. Cell number increased up to a maximum of 2.1 x 10^6^ cells/ml with a viability of 92 % after 120 h, which was quite in accordance with expectations when using a carrier concentration of 2 g/l in a batch mode process (figure 1). The growth rate µ_max_ in the interval from 24-120 h was 0.037 h^-1^ which corresponds to a doubling time of about 19 h. The handling of medium change at the beginning of the virus propagation phase was tricky but can be easily improved by using customized bags for microcarrier cultivations with media changes. The cell culture during growth phase (both populations, cells on microcarriers and in suspension) was also monitored via nexin assay determining the part of apoptotic (early and late phase) and necrotic cells. The obtained data confirm the cell count and viability data measured with the CASY TT system and microscopic observations.

The viral infection proceeded very rapidly with first signs of Influenza related cytopathic effect 24 h post infection, leading to virus yields of maximum log 3.0 HA units and 10^8.00^ TCID_50_/ml after 48 h post infection. Afterwards the infectious titer dropped slightly while HA remained stable. The virus yields achieved were 2-4-fold higher compared to standard roller bottle cultivations. For a non optimized process, these virus yields are very promising. Very likely, by finetuning of infection parameters (moi, trypsine concentration), harvest time point and increase of cell numbers before infection even higher titers are achievable.

**Figure 2:**
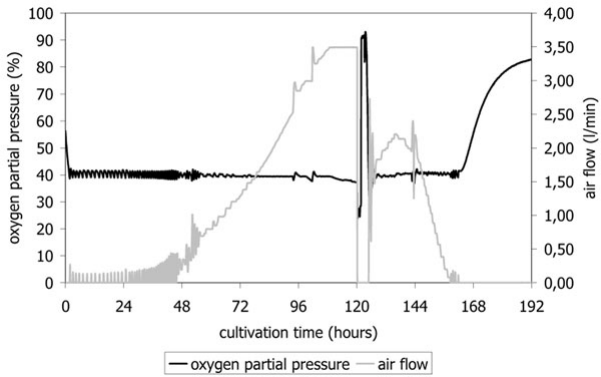
Online data from cultivation in a 50 l scale disposable stirred-tank bioreactor run for propagation of porcine Influenza A/H1N1 for oxygen partial pressure and air flow rate.

**Figure 3:**
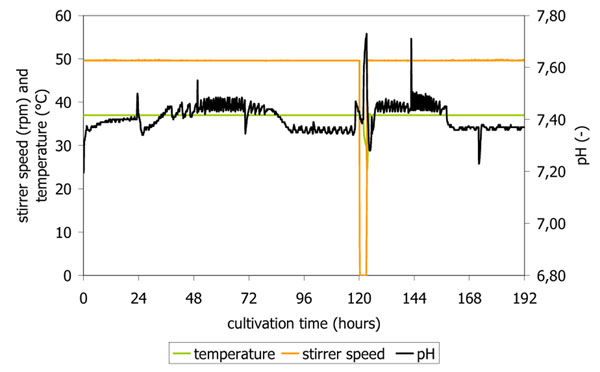
Online data from cultivation in a 50 l scale disposable stirred-tank bioreactor run for propagation of porcine Influenza A/H1N1 for temperature, stirrer speed and pH-value.

One of the most important advantages of a bioreactor system is its controlled surrounding in terms of pH and oxygen level. In this study that fact was proven very well by the good performance of the control system (figure 2 and 3). The profile for pO_2_ and air flow rate corresponds well to the cultivation curve; in the first hours of cultivation the pulsing aeration can be seen as only a few cells were in the reactor system. With increasing cell numbers a constant air flow is applied starting approx. after 48 h cultivation time leading to a maximal airflow of 3.5 l/min keeping the oxygen level constant also when higher cell concentrations are present. The virus infection led to a rapid decrease in oxygen uptake; at the end of cultivation oxygen consumption tended to be zero (figure 2). Temperature and stirrer speed control was not a problem throughout cultivation (figure 3); also control of pH value worked very well (7.40 ± 0.15).

## Conclusions

The results of this study prove a successful tech transfer for a porcine Influenza virus production process from roller bottles into a novel disposable STR bioreactor system with advances in both cell and virus yields and process control possibilities. These are important factors in near future, keeping upstream processing competitive in terms of prices, productivity and surely also from a regulatory point of view. It is a further step in order to be prepared for pandemic situations as it was seen for 2009 H1N1 occurrence. There should be no obstacles for implementing such systems into GMP surrounding. As result of the study also a lot of valuable data were generated which can be used for process validation and establishment of descriptive mathematical process models thus developing a deeper understanding of production processes for biologicals.

